# The development of biomedical engineering as experienced by one biomedical engineer

**DOI:** 10.1186/1475-925X-11-94

**Published:** 2012-12-12

**Authors:** Jonathan C Newell

**Affiliations:** 1 Department of Biomedical Engineering, Rensselaer Polytechnic Institute, 110 Eighth Street, Troy, NY, 12180, USA

**Keywords:** Biomedical engineering, Electrical impedance imaging, Impedance tomography, Impedance spectroscopy, Biomedical instrumentation

## Abstract

This personal essay described the development of the field of Biomedical Engineering from its early days, from the perspective of one who lived through that development. It describes the making of a major invention using data that had been rejected by other scientists, the re-discovery of an obscure fact of physiology and its use in developing a major medical instrument, the development of a new medical imaging modality, and the near-death rescue of a research project. The essay concludes with comments about the development and present status of impedance imaging, and recent changes in the evolution of biomedical engineering as a field.

## Correspondence

### An Electrical Engineer becomes a Biomedical Engineer

I started as an undergraduate Electrical Engineering (EE) student in the early 1960’s. But at co-op jobs in defense electronics, I decided that EE was not my chosen field. Instead, on graduation I joined the Peace Corps doing electric power engineering in the developing world. I returned to do a Masters in EE, which included a biomedical project, part of a surgical research project in trauma. With that degree, I took a draft-deferrable job in defense electronics. At age 26, I began my career as a biomedical engineer by rejoining the trauma research project at Albany (NY) Medical College (AMC). This was a project that brought computer technology to the study and care of injured patients. It consisted of a dedicated hospital room with then-state-of-the-art physiological monitoring equipment and computers (terminals to a remote mainframe) at the bedside. The project was run by a surgeon, Dr. Samuel R. Powers, Jr., and three faculty members at Rensselaer Polytechnic Institute (RPI) who provided technical expertise. It was staffed by a Surgical Fellow, a medical technician, and now an electrical engineer.

I thus began to learn the technical aspects of clinical medicine and research with on-the-job training. The equipment and techniques for physiological measurements at that time were not sophisticated or well-designed for clinical use. The whole concept of intensive care was in its infancy, and technical support from commercial equipment suppliers was weak. Also, trauma was just being recognized as an important problem by the National Institutes of Health (NIH). Specifically, what came to be called Adult Respiratory Distress Syndrome (ARDS) was just being recognized, largely due to the experience in the Vietnam War, where it was first called “Danang Lung”. Dr. Powers’ group confirmed by measuring lung volume in these patients that the primary problem involved lung collapse, which was reversed by Positive End-Expiratory Pressure (PEEP) [1]. Before those years, PEEP was thought to be dangerous – today it is a commonplace.

### Using Control Theory to Study the Lung, and a Delayed Payoff

After two years in that job, Dr. Powers advised me to get a Ph. D. in Physiology. He arranged for my admission to the Albany Medical College, and helped me apply for an NIH Special Fellowship to support my study. My dissertation evolved from a fortuitous application of my knowledge of control systems that helped discover an unknown physiological property of the lung. It had long been recognized that intrapulmonary shunt caused arterial hypoxemia. I had observed in the Trauma Unit that sometimes patients would move rapidly from a stable condition to one of high shunt and hypoxemia. I hypothesized that if arterial hypoxemia could itself make shunt worse, a positive-feedback system would result that might account for the instability. I designed an animal experiment to test this, and with the help of a fellow graduate student, Michael G. Levitzky, we went on to confirm the hypothesis and discover the underlying mechanism [2].

There was a critical “aha!” moment during the first experiment to test that hypothesis. We had placed an electromagnetic blood flow probe on the left main pulmonary artery of a dog. When we made that lung hypoxic, we expected to see a decreased blood flow, but the amplitude of the flow probe signal was unchanged. As we studied the paper polygraph record, we found by happenstance a premature ventricular contraction, followed by a long diastole with zero flow. The diastole showed that the baseline of the flowmeter had shifted, and masked a prominent retrograde flow in diastole. The peak inflow was unchanged, but the net flow was in fact reduced by that retrograde flow in diastole. I attribute the recognition of that clue to my training to be skeptical about instrumentation. Many years later, I was in the audience at an electrical impedance tomography (EIT) conference in Manchester, UK, in 2009 when Marcelo Amato from Brazil reported finding that the amplitude of the cardiac-frequency pulsatility over the lung fields was not diminished in local hypoxia. He conjectured that it was due to the mechanism we had discovered, and I was thrilled to direct him to the paper reporting our original work published 33 years earlier.

### Institutional Origins of Biomedical Engineering

I completed the Ph. D. in two years and accepted an appointment as Assistant Professor in the newly-formed Center for Biomedical Engineering at RPI in 1974. Based on my two years’ experience in the Trauma Center, I began teaching a course called ‘Clinical Engineering’, a keystone course in a new Clinical Engineering Master’s program. I also initiated a ‘Biomedical Engineering Laboratory’ course, which included the use of experimental animals, a novelty at RPI. I was also appointed Assistant Professor in the Department of Physiology at AMC, where I taught respiratory physiology to first-year medical students for 20 years, thus helping maintain my clinical contacts. And I re-joined the Trauma Center, now as an Investigator. This background probably contributed to my acceptance by the medical community, which has sometimes been an obstacle for biomedical engineers. I’ve been something of a hybrid in many ways, and I’ve found that helpful.

With these beginnings in the early 70’s, the RPI program was one of the first in the country. Over the next decade, the Center for Biomedical Engineering added faculty, student enrollment doubled, and the activity became a Department within the School of Engineering. I ran a research laboratory studying the pulmonary circulation, with NIH support, and advised a number of Ph.D. and Masters Students. The department faculty was generally involved with patient-level or organ-level studies, many in prosthesis development. Since I started in the clinical environment, I have always kept my research interests and projects closely allied with current clinical problems. I am sometimes disappointed to read theoretical studies by biomedical engineers that seem needlessly remote from clinical use.

### Impedance Imaging - an idea whose time may soon come

Around 1985 I was approached by a mathematician who was looking for a simple piece of electrical engineering data. David Isaacson, Professor of Mathematics, was making a theoretical study of Electrical Impedance Imaging, and wanted to know what noise level would be expected in measuring small voltages on the human chest at high audio frequencies. I knew nothing of Impedance Imaging, and told him I didn’t expect it to work, but I had a lab and we agreed on an experiment that could answer his question. We found an interested undergraduate, and by the end of the summer, we showed he was right, it would work, and with roughly what noise levels. From that beginning, which involved a Radio Shack audio amplifier, a hand-wound variable-tap resistor and 32 jumper wires with alligator clips, it became clear that we needed a multi-channel, computer-controlled instrument. I had worked in the Trauma Project with Prof. David Gisser, and when I approached him with the instrument design problem, he responded with four pages of hand-drawn circuits, and the project was under way [3].

The funding for this project involved a small miracle. The Trauma Unit was still functioning at this time, funded as a Program Project by NIH. At the last renewal of this Center Grant, after 22 years of support, I was Principal Investigator of one section, which proposed to use Impedance Imaging to study the lung with ARDS. The Site Visit team approved my project with a high priority and intact budget, but disapproved the whole Center. An NIH administrator named Lee Van Lenten managed to separate the EIT project from the Trauma Center, assign a new grant number, and change the institution from AMC to RPI. We had 5 years of full funding, and felt we’d caught a helicopter off the Titanic.

Electrical Impedance Imaging, as the name implies, is a technology for forming images of the interior of the body, based on organs’ varying electrical properties, using data from electrodes applied to the skin around the region of interest. Since it usually involves a single ring of electrodes and thus images a slice of the body, it is also called Electrical Impedance Tomography [4]. Some newer instruments operate across a broad frequency spectrum, and perform Electrical Impedance Spectroscopy. Most instruments measure both the real and imaginary components of the signals, and can represent the complex impedance. This relatively new field began in about 1986, with initial work by a group in Sheffield, UK, and us. The European Community funded a Concerted Action to support several laboratories in Europe, and we were declared to be “honorary Europeans” and were invited to the conferences. At that time, we were the only group in the field from the US. Now there are several.

The technology works by applying small, high-frequency currents (or voltages) to many (usually 16 to 256) electrodes, and measuring the resulting voltages (or currents). Most of the European systems used a single current source and multiple voltage measurements, which is relatively easy to build, and works fairly well. We elected to use as many current sources as we had electrodes (32) and measure voltage on all these electrodes. This system has theoretical advantages over single-source systems, but is a lot harder and more expensive to build [3,5]. Both designs have existed since around 1988, and neither has replaced the other to this day. The other important design criterion for multiple-source systems is whether to use voltage or current sources. Current sources are less sensitive to high-spatial-frequency noise, but are difficult to implement for broadband applications, and are intolerant of open electrode connections. We used current sources for our single-frequency instruments, but moved to voltage sources for the broadband ACT 4. Prof. Gary Saulnier has since patented a current source design that is suitable for use in broadband, multiple source applications.

For over two decades, my colleagues and I have been at the cutting edge of this technology, having to define the problems before we could solve them. It is an exhilarating experience to be part of a small but world-wide community of workers trying to invent something completely new. The caveats for those who would pursue such a path are many, however. There needs to be a balance between developing a promising idea because it seems to have potential, and identifying an application for which it is truly useful. Clinical utility is a high hurdle. But if you don’t have a vision, it’s hard to do anything.

Many applications of EIT have been proposed, and tested for feasibility. In Russia, a system is being marketed for breast cancer detection [6]. But by 2012, most other applications have languished, and the only other application that appears ready to become commercial is the monitoring of cardiopulmonary function in acute care patients. An instrument is being tested by Draeger, based on a 16-electrode design developed in Germany [7]. Recently, a major US manufacturer of medical instruments has hired three of our former students, and obtained an NIH grant to develop a commercial clinical instrument. We are collaborating with that effort, which also has clinical colleagues at Columbia University/NY Presbyterian Medical Center in New York.

**Figure 1:**
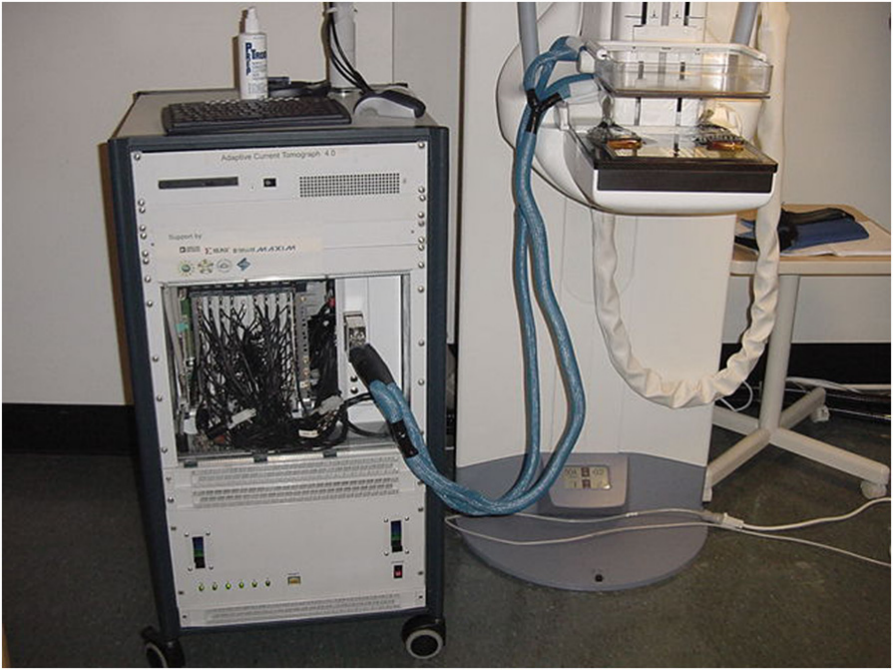
The latest ACT 4 instrument installed adjacent to a Tomosynthesis machine.

Throughout these developments, our EIT project has had intermittent funding from NIH, and 10 years of support through an Engineering Research Center from the National Science Foundation. We have spent around $5 million, and built four generations of instrument. The last was designed by Dr. Gary Saulnier of RPI, and is a 60-electrode machine operating from 3 kHz to 1 MHz at 7 frames/sec. It has two plates with radiolucent electrodes that are placed on a 3-D mammography machine, allowing simultaneous 3-D mammograms and 3-D EIT images to be produced, in-register. We have collaborated with Dr. Daniel Kopans at Massachusetts General Hospital to study breast cancer patients, with results that are still being analyzed (Figure 1).

In the 26 years since our first publication, the EIT project has helped educate over 100 students, including several post-doctoral fellows. And four faculty members from universities in Asia have visited for several months.

### Biomedical Engineering - Challenges and Prospects

While this instrumentation development project was taking place, the field of Biomedical Engineering was changing radically. The discipline of Biomedical Engineering started with a few random, isolated individuals who took an interest in some aspect of health care and clinical medicine. They invented devices to solve specific clinical problems, such as the pacemaker, defibrillator, electrocardiogram (EKG) monitors and thermodilution cardiac output detectors. The *ad-hoc* nature of the field is exemplified by the fact that defibrillators were on the market for many years before defibrillator testers came along. When they did, it was discovered that all defibrillators delivered only about 80% of their stored energy. That finding raised the question of which energy to report to the operator – the stored energy, which is what all early machines reported, or the more clinically relevant delivered energy. For many years, machines showed both. Today, you can buy an Automatic External Defibrillator (thanks largely to the work of Dr. Fred Chapman, one of my students) for a few hundred dollars, and keep it in your home. Another interesting example is the Pulse Oximeter. It was invented in Japan, but the inventor never filed a US patent. Many companies scrambled to enter that market, and the price fell from around $3000 for the early versions to a device you can buy for yourself for less than $40 [8]. The invention of that device holds a lesson for any future device designer. For many years, several companies had tried to extract oxygen saturation information from light shined through tissue. But the presence of other pigments such as myoglobin made the problem complex, and the signals were hard to process because they varied a lot with the heartbeat. Hewlett Packard sold a machine with 8 different light wavelengths to try to separate out just the blood signal. A man in Japan named Aoyagi realized that those pulse-related signals, which stymied everyone else, actually contained the solution. The only thing varying was the arterial blood. If he analyzed only the changes in light absorption, he only needed two wavelengths. He used the signal that everybody else tried to work around or eliminate.

Simultaneously with these changes, the technology of medical imaging expanded from straightforward X-Rays to Nuclear Magnetic Resonance (now called Magnetic Resonance Imaging because “nuclear” sounded scary), Computed Tomography, and Positron Emission Tomography. There was always something of a separation between these large instruments, marketed to radiology departments, and general BME, whose domain was the entire medical care community. But each informed the other to some extent.

With the formation of organizations such as the Association for the Advancement of Medical Instrumentation, which went on to organize Biomedical Engineering Technicians, the field began to mature. I believe a critical role was played by the Veterans’ Administration Hospital system, which was quick to introduce the position of Clinical Engineer to its system. Another early important step was made by Dr. Joel Nobel, who founded the Emergency Care Research Institute [9], which began evaluating medical instruments and soon began publishing a journal to support the work of Clinical Engineers. ECRI has become a clearing house for clinical instrumentation reports of successes and failures. If you believe that feedback can improve the performance of an engineering system, you can appreciate the contribution of Dr. Nobel.

### Quo Vadem?

These activities and ones like them defined the field of Biomedical Engineering from the late 1960’s through the late 1990’s. At around that time, the instrumentation field became, in many respects, a mature technology with developments better characterized as incremental rather than groundbreaking. Between the late 1990’s and mid-2000’s, the bulk of innovative activity moved to cellular- and molecular-level studies generally characterized as Tissue Engineering. As the basic biology became much better understood, cell culture techniques were refined and taken up by Bioengineers, and the thrust became to design and build replacement tissues. Starting with skin, but soon including blood vessels, neural tissue, and endocrine functions such as pancreatic beta cells, the nature of the expertise needed by a Biomedical Engineer took a radical change. Basic cellular level biology, including genomics and nanotechnology have all nearly merged with biomedical engineering, and the engineers have taken on the practical goals of building (growing) something useful.

So in recent years, cell culture and other new techniques have become critical parts of the curriculum for Bioengineering. This clearly promising development will have important medical applications. But we should not forget that there remain immediate clinical needs to be met while the longer term solutions are developed. The tragedy of medicine is that patients continue to become ill before we’ve learned how to cure them. An eclectic approach to education still seems best.

As I try to extract from this story any message useful to others, the point that seems to me most clear is that I’m a hybrid who does best working at interfaces. I’ve never known as much about my colleagues’ fields as they do, but by working with colleagues in different fields on a project that needs expertise in those fields, we’ve gotten much further than any of us could have gone alone. I’ve often found that the most useful things I know are things I wasn’t “supposed” to know. So in teaching Biomedical Engineering, I always tried to teach a diversity of skills, and to teach students to be able to communicate clearly with folks in other disciplines. That’s not a new message, but I hope to have given it weight by example.

## Abbreviations

EE: Electrical Engineering; AMC: Albany (NY) Medical College; RPI: Rensselaer Polytechnic Institute; NIH: National Institutes of Health; ARDS: Adult Respiratory Distress Syndrome; PEEP: Positive End-Expiratory Pressure; EIT: Electrical Impedance Tomography; EKG: Electrocardiogram; ECRI: Emergency Care Research Institute.

## Competing interests

I have no competing interests in this work.
